# ClusterBootstrap: An R package for the analysis of hierarchical data using generalized linear models with the cluster bootstrap

**DOI:** 10.3758/s13428-019-01252-y

**Published:** 2019-05-14

**Authors:** Mathijs Deen, Mark de Rooij

**Affiliations:** Institute of Psychology, Methodology and Statistics Unit, Leiden University, Wassenaarseweg 52, 2333 AK Leiden, The Netherlands

**Keywords:** Clustered data, Hierarchical data, Generalized linear models, Cluster bootstrap

## Abstract

In the analysis of clustered or hierarchical data, a variety of statistical techniques can be applied. Most of these techniques have assumptions that are crucial to the validity of their outcome. Mixed models rely on the correct specification of the random effects structure. Generalized estimating equations are most efficient when the working correlation form is chosen correctly and are not feasible when the within-subject variable is non-factorial. Assumptions and limitations of another common approach, ANOVA for repeated measurements, are even more worrisome: listwise deletion when data are missing, the sphericity assumption, inability to model an unevenly spaced time variable and time-varying covariates, and the limitation to normally distributed dependent variables. This paper introduces ClusterBootstrap, an R package for the analysis of hierarchical data using generalized linear models with the cluster bootstrap (GLMCB). Being a bootstrap method, the technique is relatively assumption-free, and it has already been shown to be comparable, if not superior, to GEE in its performance. The paper has three goals. First, GLMCB will be introduced. Second, there will be an empirical example, using the ClusterBootstrap package for a Gaussian and a dichotomous dependent variable. Third, GLMCB will be compared to mixed models in a Monte Carlo experiment. Although GLMCB can be applied to a multitude of hierarchical data forms, this paper discusses it in the context of the analysis of repeated measurements or longitudinal data. It will become clear that the GLMCB is a promising alternative to mixed models and the ClusterBootstrap package an easy-to-use R implementation of the technique.

## Introduction

In behavioral research, various techniques are being used to analyze hierarchical data. Some examples of hierarchical data (sometimes called nested or clustered data) are children that are observed within the same classes or patients in a clinical trial that are being treated at the same department. When analyzing such data, it is paramount to take into consideration the fact that children within the same classes are more alike than children from different classes, and that patients within the same department are likely to be more alike than patients from different departments. Data are also hierarchical when there are repeated measurements within persons. The repeated measurements within a person tend to be correlated, where this is not necessarily the case for the observations from different persons. For the analysis of repeated measurements, the repeated measures analysis of variance (RM-ANOVA) is popular, because this method is well understood by experimental psychologists and often taught to undergraduate psychology students. Moreover, popular statistical textbooks (e.g., Brace et al.,, 2016; Pallant, 2013) advocate the use of this technique, perhaps because it is part of the ANOVA framework that is at the core of introductory statistical courses. There are, however, some downsides to the use of RM-ANOVA, such as its incapability to use time-varying explanatory variables and a non-factorial (e.g., unevenly spaced) time variable, as well as a loss of power when confronted with missing data, because RM-ANOVA completely removes a case when one measurement occasion is not accounted for. Also, when the dependent variable is not normally distributed, RM-ANOVA is inappropriate.

There are several alternatives to RM-ANOVA, such as generalized linear mixed models (GLMMs), also known as hierarchical linear models, multilevel models, or variance components models (Goldstein, 1979; Raudenbush & Bryk, 2002; Verbeke & Molenberghs, 2009) and generalized estimating equations (GEE; Liang & Zeger, 1986; Hardin & Hilbe, 2003). A third alternative is to use generalized linear models with the cluster bootstrap (GLMCB; Davison & Hinkley, 1997; Field & Welsh, 2007; Harden, 2011; Sherman & LeCessie, 1997). Unlike RM-ANOVA, these techniques can handle the presence of missing data (to some extent), a non-normal dependent variable or a non-factorial time variable. McNeish et al., (2017) recently highlighted some advantages of the GEE and GLMCB approach in comparison to GLMMs. Below, these techniques will be discussed in more detail. Since they can all be seen as extensions of the framework of generalized linear models, these will be discussed first.

### Generalized linear models

Many problems can be written as a regression problem. When we have a single response variable *Y* with observations *y*_*i*_, *i* = 1,…,*n* and a set of predictor variables *x*_*i*1_,*x*_*i*2_,…,*x*_*ip*_, the standard multiple linear regression model is

$$ \begin{array}{@{}rcl@{}} y_{i} &=& \alpha +\beta_{1} x_{i1}+\beta_{2} x_{i2}+\beta_{3} x_{i3}+\ldots+e_{i}\\ &=& \alpha + \sum\limits_{j} \beta_{j} x_{ij} +e_{i}. \end{array} $$

where *e*_*i*_ are residuals. In standard applications (in cross-sectional data analysis), these residuals are assumed to be normally distributed with mean zero and constant variance ($e_{i} \sim N(0,{\sigma ^{2}_{e}})$). For categorical predictor variables, dummy variables are created.

Generalized linear models (GLMs; McCullagh and Nelder, 1989) generalize the regression model in two aspects: (a) The dependent variable may have another distribution than the normal; and (b) the dependent variable is not described itself (by a linear model) but a function of the response variable is. GLMs then have three components:

1. *Random component*: The probability density function for the response variable must be from the *exponential family*, that has the form
  $$ \begin{array}{@{}rcl@{}} f(y_{i};\theta_{i},\phi) = \exp\left( \frac{y_{i}\theta_{i}-b(\theta_{i})}{a(\phi)}+c(y,\phi)\right), \end{array} $$
  for the natural parameter *𝜃*_*i*_, dispersion parameter *ϕ*, and functions a(⋅), b(⋅), and c(⋅). Special cases of this family are, among others, the normal distribution, the binomial distribution, and the Poisson distribution (see McCullagh & Nelder 1989, for proofs).
2. *Systematic component*: This is the linear part of the model
  $$ \begin{array}{@{}rcl@{}} \eta_{i} &=& \alpha + \sum\limits_{j} \beta_{j} x_{ij}. \end{array} $$
3. *Link function*: A function that links the expectation *E*(*y*_*i*_) = *μ*_*i*_ to the systematic component *η*_*i*_.
  $$ \begin{array}{@{}rcl@{}} g(\mu_{i})=\eta_{i} = \alpha + \sum\limits_{j} \beta_{j} x_{ij}. \end{array} $$
  Main examples are the identity link, *g*(*μ*) = *μ* for linear regression; the logit transformation $g(\mu ) = \log (\frac {\mu }{1-\mu })$, which is used in logistic regression; and the log transformation *g*(*μ*) = log(*μ*) that is appropriate for count data.

For the remainder of this paper, we will be especially interested in continuous and dichotomous dependent variables with the above-mentioned link functions. For a continuous variable with an identity link, we thus have

$$ \begin{array}{@{}rcl@{}} \mu_{i} = \alpha + \beta_{1} x_{i}, \end{array} $$

so that the expected value given *x*_*i*_ = 0 equals *α* and with every unit increase of *x* the response increases by *β*_1_. For binary response variables, *μ*_*i*_ indicates the probability of one of the two categories of the response variable and with a logistic link we have

$$ \begin{array}{@{}rcl@{}} \log\left( \frac{\mu_{i}}{1-\mu_{i}}\right) = \alpha + \beta_{1} x_{i}, \end{array} $$

so that the expected log odds given *x*_*i*_ = 0 equals *α* and with every unit increase of *x* the log odds increases by *β*_1_.

### Generalized linear mixed models

GLMMs can be regarded as an extension of the GLM framework (Gelman & Hill, 2007): there is an outcome variable and there are usually several explanatory variables. GLMMs are also widely known as multilevel models (Hox et al., 2017; Snijders & Bosker, 2012) and hierarchical generalized linear models (Raudenbush & Bryk, 2002). In the context of longitudinal data, there usually is a variable among the explanatory variables that represents time. This implies that data are arranged in a long format: every observation (i.e., each timepoint) of every subject occupies a single row in the dataset. The fact that each subject (the so-called level-2 unit) now has multiple observations (level-1 units) in the dataset implies that the observations are not independent of each other. The violation of the independence assumption of GLM requires the regression model to be extended. This extension of the linear model lies in the addition of so-called random effects. Usually, a random intercept and a random slope for the time-varying level-1 variable (e.g., time) are incorporated, with mean vector **0** and a covariance matrix **Σ**.

#### Omission of random effects

The GLMM is most efficient when the random part of the model is specified correctly. They are, however, not observed directly, which makes it impossible to assess whether the true random effects structure is modeled (Litière et al., 2007, 2008).

Several papers have investigated the consequences of omitting a random effect. Tranmer and Steel (2001) demonstrate that, in a hypothetical three-level LMM, the complete omission of a level leads to redistribution of the variance in the ignored level into the lower and higher level of the modeled two-level LMM, subsequently. Moerbeek (2004) and Berkhof and Kampen (2004) elaborate on these findings, and show that for unbalanced designs (in a longitudinal context, i.e., a non-fixed number of repeated measurements), the omission of a level (Moerbeek, 2004) or only including a level partially (by omitting either the random intercept or the random slope; Berkhof & Kampen, 2004) may lead to incorrect conclusions based upon *p* values. Van den Noortgate et al., (2005) conclude that standard errors for fixed effects on the ignored level and adjacent level(s) are affected the most. The mentioned studies all focus on LMMs with more than two levels, and all but one (Berkhof and Kampen, 2004) focus on the complete omission of one or several levels.

For two-level data, Lange and Laird (1989) show that, in a balanced and complete setting, for linear growth curve models where the true error covariance structure implies more than two random effects, a model including only two random effects leads to unbiased variance estimates for the fixed effects. Schielzeth and Forstmeier (2009) and Barr et al., (2013) discuss the common misconception that models with only a random intercept are sufficient to satisfy the assumption of conditional independence, even when random slope variation is present. Schielzeth and Forstmeier (2009) conclude that one should always incorporate random slopes as well, as long as this does not lead to convergence problems. Barr et al., (2013) recommend using as many random effects as possible. Lastly, outside the framework of LMM, Dorman (2008) shows that type I errors inflate as the variance partition coefficient (VPC; Goldstein et al., 2002, often and hereafter referred to as the intraclass correlation of the random effect, ICC) that is not accounted for, increases.

### Generalized estimating equations

In GEE (Liang and Zeger, 1986), simple regression procedures are used for the analysis of repeated measurements data. The procedure adapts the standard errors by using a robust sandwich estimator (Liang & Zeger, 1986), adjusting the standard errors when the true variance is inconsistent with the working variance guess. For a more thorough description of the sandwich estimator, we refer to Agresti (2013, Chapter 14). GEE is closely related to GLMCB, as both specify marginal models. GEE is, however, built on asymptotic results. For small samples, it is questionable whether the procedure really works well (e.g., Gunsolley et al.,; McNeish & Harring, 2017; Yu & de Rooij, 2013). In GEE, a working correlation form has to be chosen to model the correlation between repeated measurements. Common choices for this working correlation include the exchangeable, the autoregressive, the unstructured, and the independent correlation structure. Note that the latter assumes no correlation between repeated measurements, which leads to regression estimates that are identical to those of GLM. For an overview of these correlation structures, see Twisk (2013, Chapter 4). Many papers have been written about the choice of working correlation form. Some conclude that the estimates are more efficient when the working form is closer to the true form (Crowder, 1995). Others show that simple working forms are often better (Lumley, 1996; O’Hara Hines, 1997; Sutradhar & Das, 1999). Furthermore, if one is interested in effects with time-varying explanatory variables, one should be very careful about the choice of working correlation form (Pepe & Anderson, 1994).

### Generalized linear models with the cluster bootstrap

Often statistical inference and stability are assessed using asymptotic statistical theory assuming a distribution for the response variable. In many cases, however, such asymptotic theory is not available or the assumptions are unrealistic and another approach is needed. Nonparametric bootstrapping (Efron, 1982; Efron & Tibshirani, 1993; Davison & Hinkley, 1997) is a general technique for statistical inference based on building a sampling distribution for a statistic by resampling observations from the data at hand. The nonparametric bootstrap draws at random, with replacement, *B* bootstrap samples of the same size as the parent sample. Each of these bootstrap samples contains subjects from the parent sample, some of which may occur several times, whereas others may not occur at all. For regression models (GLMs), we can choose between randomly drawing pairs, that is both the explanatory and response variables, or drawing residuals. The latter assumes that the functional form of regression model is correct, that the errors are identically distributed and that the predictors are fixed (Davison & Hinkley, 1997; Fox, 2016). For the ClusterBootstrap procedure, random drawing of pairs is chosen as the sampling method to avoid the dependency upon these assumptions.

For hierarchical or clustered (e.g., longitudinal, repeated measurement) data, in order to deal with the within- individual dependency, the sampling is performed at the individual level rather than at the level of a single measurement of an individual (Davison & Hinkley, 1997). This implicates that when a subject is drawn into a specific bootstrap sample, all the observations from this subject are part of that bootstrap sample. The idea behind this is that the resampling procedure should reflect the original sampling procedure (Fox, 2016, p. 662-663). For repeated measurements, the researcher usually recruits subjects, and within any included subject, the repeated measurements are gathered. In other words, the hierarchy of repeated measurements within subjects that is present in the original data should be and is reflected within each bootstrap sample. Because the observations within a single subject are usually more closely related than observations between different subjects, the bootstrap samples obtained by using such a clustered sampling scheme are more alike, thereby reducing the variability of the estimates. Moreover, in each bootstrap sample, the dependency among the repeated measurements is present. In repeated measurements, this dependency is usually of an autoregressive kind; this autoregressive structure is still present in each bootstrap sample due to the drawing of clusters of observations (i.e., all observations from the subjects being drawn). Using this sampling approach with generalized linear models is referred to as generalized linear models with the cluster bootstrap. The term ”cluster” here refers to observations being dependent upon each other in a hierarchical way (e.g., repeated measurements within persons, children within classes) and has no relation to cluster analysis, where the aim is to find clusters of observations with similar characteristics.

Clustered resampling has been investigated scarcely since the mid-1990s. Field and Welsh (2007) show that the cluster bootstrap provides consistent estimates of the variances under different models. Both Sherman and LeCessie (1997) and Harden (2011) show that the cluster bootstrap outperforms robust standard errors obtained using a sandwich estimator (GEE) for normally distributed response variables. Moreover, Sherman and LeCessie (1997) show the potential of the bootstrap for discovering influential cases. In their simulation study, Cheng et al., (2013) propose the use of the cluster bootstrap as an inferential procedure when using GEE for hierarchical data. They show, theoretically and empirically, that the cluster bootstrap yields a consistent approximation of the distribution of the regression estimate, and a consistent approximation of the confidence intervals. One of the working correlation forms in their Monte Carlo experiment is the independence structure, which, as mentioned earlier, gives parameter estimates that are identical to the ones from GLM, and when integrated in a cluster bootstrap framework, are identical to the estimates from GLMCB. In the cases of count and binary response variables, they show that the cluster bootstrap outperforms robust GEE methods with respect to coverage probabilities. For Gaussian response variables, the results are comparable. Both Cameron et al., (2008) and McNeish (2017) point out that for smaller sample sizes, GLMCB may be inappropriate because the sampling variability is not captured very well (i.e., it tends to remain underestimated) by the resampling procedure. Feng et al., (1996), however, show that when the number of clusters is small (ten or less), the cluster bootstrap is preferred over linear mixed models and GEE when there are concerns regarding residual covariance structure and distribution assumptions.

Despite the support for GLMCB being a strong alternative to more common methods like GLMM and GEE, there is still hardly any software readily available for researchers to apply this method. In the present paper, we introduce ClusterBootstrap (Deen and De Rooij, 2018), which is a package for the free software environment R (R Core Team, 2016). After discussing the algorithm involved, we will demonstrate the possibilities of the package using an empirical example, applying GLMCB in the presence of a Gaussian and a dichotomous dependent variable. Subsequently, GLMCB will be compared to linear mixed models in a Monte Carlo experiment, with prominence given to the danger of incorrectly specifying the random effects structure.

## Algorithm

### Balanced bootstrap

The balanced bootstrap can be used to ensure that every individual appears exactly *B* times in the bootstrap samples, in contrast to randomly drawing bootstrap samples from the parent sample. Davison and Hinkley (1997) show that the balanced bootstrap results in an efficiency gain.

For unbalanced longitudinal data, where some subjects have more measurements than others, the balanced bootstrap ensures that the average size of the bootstrap samples equals the (subject) sample size *N*. In the balanced bootstrap, rather than simply drawing at random, a matrix is made with *B* copies of the numbers 1 to *N*. This matrix is vectorized, randomly shuffled, and turned back into a matrix of size *N* × *B* (Gleason, 1988). Each of the columns of this latter matrix gives the indices of a single bootstrap sample.

### Confidence intervals

The parameters of interest in the current context are the regression weights, the *β*’s. Various types of stability measures can be obtained for these parameters from the bootstrap. We will discuss the parametric, the percentile, and the bias-corrected and accelerated confidence intervals.

#### Parametric interval

The bootstrap normal-theory interval assumes that the statistic *β* is normally distributed, and uses the bootstrap samples to estimate the sampling variance. Let $\bar {\beta }^{*}$ denote the average of the bootstrapped statistics *β*^∗^, that is, $\bar {\beta }^{*}={\sum }_{b=1}^{B}\beta _{b}^{*}/B$, where $\beta ^{*}_{b}$ is the estimate of *β* in the *b*-th bootstrap sample $\mathcal {S}^{*}_{b}$. The sampling variance of *β* is ${\sum }_{b=1}^{B}(\beta _{b}^{*}-\bar {\beta }^{*})^{2}/(B-1)$. The standard deviation ($\sqrt {\text {Var}(\beta ^{*})}$) is an estimate of the standard error of *β*, SE(*β*). A 95% confidence interval based on normal theory is

$$ \hat{\beta}\pm 1.96\mathrm{\widehat{SE}}(\beta^{*}), $$

where $\hat {\beta }$ is the estimate from the original sample.

#### Percentile interval

This approach uses the empirical distribution of $\beta _{b}^{*}$ to form a confidence interval for *β*. Therefore, first, rank order the estimates from the bootstrap samples $\beta _{(1)}^{*},\beta _{(2)}^{*},\ldots ,\beta _{(B)}^{*}$, so $\beta _{(1)}^{*}$ is the smallest regression weight obtained and $\beta _{(B)}^{*}$ the largest. The 100(1 − *α*)% percentile interval is then specified as $[\beta _{B\times \frac {\alpha }{2}}^{*},\beta _{B\times (1-\frac {\alpha }{2})}^{*}]$. With *B* = 5000 bootstraps, a 95% percentile confidence interval is given by $[\beta _{(125)}^{*},\beta _{(4875)}^{*}]$.

#### Bias-corrected and accelerated interval

The coverage of the percentile approach can be improved by implementing the bias-corrected and accelerated (BCa) interval. The BCa method uses a bias correction factor ($\hat {z}_{0}$) and an acceleration factor ($\hat {a}$) to correct for asymmetry among the bootstrap estimates and the normalized rate of change of the standard error of $\hat {\beta }$ with respect to the true parameter value *β*, respectively (Efron & Tibshirani, 1993; Yu & de Rooij, 2013). For a 100(1-*α*)% BCa interval of $\hat {\beta }$, the BCa method defines the endpoints as

$$ \begin{array}{@{}rcl@{}} \hat{\beta}_{lower}^{*} &=& B \times {\Phi}\left[\hat{z}_{0}+\frac{\hat{z}_{0}+z_{\alpha/2}}{1-\hat{a}(\hat{z}_{0}+z_{\alpha/2})}\right] \\ \hat{\beta}_{upper}^{*} &=& B \times {\Phi}\left[\hat{z}_{0}+\frac{\hat{z}_{0}+z_{1-\alpha/2}}{1-\hat{a}(\hat{z}_{0}+z_{1-\alpha/2})}\right], \end{array} $$

with Φ(⋅) being the standard normal cumulative distribution function. The bias-correction factor $\hat {z}_{0}$ obtained using the proportion of bootstrap estimates less than the original estimate is defined as

$$ \hat{z}_{0} = {\Phi}^{-1}\left[\frac{\#_{b=1}^{B}(\hat{\beta}^{*}<\hat{\beta})}{B}\right], $$

and the acceleration factor $\hat {a}$ as

$$ \hat{a} = \frac{\sum\limits_{i=1}^{n} (\hat{\beta}_{(\cdot)}-\hat{\beta}_{(-i)})^{3}}{6\left[\sum\limits_{i=1}^{n} (\hat{\beta}_{(\cdot)}-\hat{\beta}_{(-i)})^{2}\right]^{\frac{3}{2}}}, $$

where $\hat {\beta }_{(-i)}$ is the estimate for $\hat {\beta }$ with all measurements for subject *i* removed, and

$$ \hat{\beta}_{(\cdot)} = \frac{1}{n}{\sum}_{i=1}^{n} \hat{\beta}_{(-i)}. $$

This resembles the so-called jackknife (Efron, 1982; Efron & Tibshirani, 1993), albeit in a ”clustered” way (i.e., removing all observations within subject *i* instead of removing single observations).

## Motivating example

As an example, we will use data from a study by Tomarken et al., (1997), which are used by Singer and Willett (2003, pp. 181-188) in their textbook on longitudinal data analysis. The aim of this study was to evaluate the effectiveness of additional antidepressant medication for outpatients with a major depressive disorder. The data consist of repeated measurements in 73 participants during the first week of the study, in which they received either a treatment or a placebo drug and were asked to fill in a mood diary three times a day. In the current data, positive affect is the dependent variable, and treatment condition, time (in days), and their interaction are the independent variables. Participants were regarded as compliant when at least 16 of the 21 measurements were completed, which was not the case for two participants who filled in two and 12 diary entries.

## R package: ClusterBootstrap

### Preparation

The latest stable version of ClusterBootstrap can be installed from the CRAN repository. The package can be loaded using

> library("ClusterBootstrap")

### Input and exploration

Data needs to be arranged in a long format: every observation is represented in a single row. A unique identifier distinguishes the clusters (e.g., a subject that has multiple measurement occasions) from one another. This format is also appropriate for GLMM and GEE. The current version of ClusterBootstrap uses the glm function that is part of the base install of R. This makes available the binomial, Gaussian, gamma, inverse Gaussian, Poisson, quasibinomial and quasi-Poisson distributions, as well as the quasi option for a user-defined variance function. The distributions that have been tested intensely thus far are the Gaussian and the binomial. Our example data is included in the package and can be loaded using

> data(medication)

To get an idea of what the data look like, we can look at the first five measurement occasions of participants 1 and 10:

> medication[c(1:5,21:25),]id treattimepos111 0.0000 106.7211 0.3333 100.0311 0.6667 100.0411 1.0000 100.0511 1.3333 100.021100 0.0000 243.322100 0.3333 226.723100 0.6667 236.724100 1.0000 183.325100 1.3333 166.7 showing the cluster identifier (id), a between-subjects variable (treat), a variable varying within subjects (time), and a variable pos, which is the dependent variable in our analysis.

### Analysis

The main analysis can be carried out using the clusbootglm function in the following way:

> set.seed(1)> model.1 <- clusbootglm(pos ∼ treat⋆time,data = medication,clusterid = id) Other arguments that can be specified are B for the number of bootstrap samples, family for the error distribution, confint.level for the level of the confidence interval, and n.cores for the number of CPU cores to be used in parallel to speed up the computations.

#### Parallel computing

For parallel computing, ClusterBootstrap depends on the parallel package, using the random number generator of L’Ecuyer (1999) without a predefined seed as a subsequent to the seed that was initially set by the user. This gives certainty to the reproducibility of the findings when the user sets the seed prior to calling the clusbootglm function. If one wishes to use multiple CPU cores, it is advised (especially for Windows and Sparc Solaris operating systems) to leave at least one processor unused. The number of available processors can be requested by parallel::detectCores(). By not making use of forking, which is not available for Windows, the implementation of parallel processing is identical for all operating systems, as is the generated output given a certain seed.

### Investigating the output

The function summary can be used to get an overview of the parameter estimates and their dispersion characteristics.

> options(digits= 3)> summary(model.1)Call:clusbootglm(model = pos ∼ treat ⋆ time,data = medication, clusterid = id)

Estimate Std.error CI 2.5% CI 97.5%(Intercept) 167.259.09150.48186.52treat-6.3312.27-31.5016.73time-2.051.46-4.601.29treat:time5.682.211.5210.26---95% confidence interval using bias correctedand accelerated cluster bootstrap intervals

The summary function returns parameter estimates, the bootstrap standard deviation, and, by default, the confidence interval at the level that was specified in the analysis. The standard interval method is BCa, though this can be altered using the interval.type argument in the summary function.

The confint function lets the user change the level of the confidence interval post hoc (i.e., the bootstrap procedure need not to be performed again). For example, to get a 90% parametric confidence interval level of the time and the treat⋆time parameters, one can use

> confint(model.1, level=.90,parm=c("treat","treat:time"),interval.type="parametric")5%95%treat-26.59 13.77treat:time2.039.32

To extract the parameter estimates from the model, the function coef can be used, with the option to choose either the bootstrap coefficient means (which is the default) or the coefficients from the GLM that was fitted on the original data:

> coef(model.1, estimate.type="GLM")GLM(Intercept) 167.26treat -6.41time -2.04treat:time 5.68 Based on the regression parameters and their confidence intervals, our conclusion would be that although there are no overall differences between the treatment conditions regarding their positive mood and there is no main effect for the time variable, there is a difference between the two treatment groups regarding their effects over time. Assuming the nonsignificant main effects are zero and assuming the treatment group is coded 1 and the placebo group is coded 0, the significant estimate of 5.68 exclusively for the treatment group would lead one to conclude that the treatment group gains positive mood over time, where the placebo group does not.

The bootstrapped covariance matrix of the parameter estimates can be obtained using the estimates from the individual bootstrap samples:

> cov(model.1$coefficients)(Intercept)treattime treat:time(Intercept)82.69 -82.98 -7.887.81treat-82.98 150.518.06-12.27time-7.888.062.15-2.13treat:time7.81 -12.27-2.134.90

The covariance matrix can be interpreted easily in the light of the bootstrap procedure. For example: within the 5000 bootstrap samples, there seems to be a positive relation between the estimated values of treatment and time ($r \approx -7.88/\sqrt {150.51 \times 2.15} \approx .44$) and a negative association between the estimated coefficients of treatment and the interaction term (*r* ≈−.45).

### Checking bootstrap samples with issues

An issue that might evolve in any bootstrap procedure is that the statistics of interest cannot be computed in some of the bootstrap samples. In the context of GLM, this might occur when there is complete or quasi-complete separation. For example, complete separation occurs in logistic regression when a hyperplane can pass through the explanatory variable space in such a way that all cases with *y*_*i*_ = 0 are on one side of the hyperplane and all cases with *y*_*i*_ = 1 are on the other side (Agresti, 2013, p. 234). Quasi-complete separation refers to a weaker form of this situation (i.e., there is an almost perfect discrimination of the outcome variable by the explanatory variable space). Another potential issue is when there is no variation in the outcome variable. In logistic regression, for example, the chance of the absence of variation in the outcome variable in any of the bootstrap samples increases when the count of either one of the outcome categories decreases. To simulate such a situation, we can split the pos variable from the medication data at the 99th percentile, and use the dichotomous resultant as an outcome in a logistic regression with the cluster bootstrap:

> medication$pos_dich <- with(medication,ifelse(pos>quantile(pos,.99),1,0))> set.seed(1)> model.2 <- clusbootglm(pos_dich ∼ treat⋆time,data = medication,clusterid = id,family = binomial)

Now, when the summary function is invoked, there is an extra line, indicating a problem in 30 bootstrap samples:> summary(model.2)Call:clusbootglm(model = pos_dich ∼ treat ⋆ time,data = medication, clusterid = id,family = binomial)

Estimate Std.error CI 2.5% CI97.5%(Intercept)-5.3573.851-21.57-2.812treat-2.5887.161-20.234.791time-0.2910.648-2.160.733treat:time0.3480.993-1.082.983---95% confidence interval using bias correctedand accelerated cluster bootstrap intervalsThere were 30 bootstrap samples which returnedat least one NA

We can investigate which bootstrap samples are having issues:

> model.2\$samples.with.NA.coef[1] 13 431 517 622 704 1009[7] 1334 2244 2249 2277 2302 2328[13] 2388 2406 2519 2579 2662 2935[19] 3180 3675 3927 4023 4143 4458[25] 4484 4562 4593 4656 4777 4887

If we wish to further investigate any of these bootstrap samples (e.g., the first one, being bootstrap sample 13), we can obtain the corresponding dataset:

> clusbootsample(model.2,13)id treattime pos pos_dich100 2810.000 1070101 2810.333 1200102 2810.667 1270103 2811.333 1000104 2811.667 1470105 2812.000 1270...<< 1254rows omitted>>...60914115.00177061014115.33280061114115.67167061214116.00230061314116.33187061414116.672800

Summing the fifth column of this data frame tells us that all the values on the dichotomous outcome are zero, indicating no variation in the outcome variable. In any case, the resulting data frame could subsequently be used in a regular application of the glm() function to obtain relevant information about the issue at hand or, for example, to obtain the parameter estimates:

> glm(pos_dich ∼ treat⋆time,data = clusbootsample(model.2,13),family = binomial)Call:glm(formula = pos_dich ∼ treat⋆time,family = binomial,data = clusbootsample(model.2,13))Coefficients:(Intercept)treattimetreat:time-2.66e + 012.52e-13-1.24e-27-5.59e-14Degrees of Freedom: 1265 Total (i.e. Null);1262 ResidualNull Deviance:0Residual Deviance: 7.34e-09AIC: 8Warning message:glm.fit: algorithm did not converge

For each of the coefficients, we can also obtain the amount of NA s in our bootstrap samples:

> model.2$failed.bootstrap.samples(Intercept)treattimetreat:time30303030

In this example, the number of NA s is equal for all coefficients, which might indicate 30 bootstrap samples have some overall convergence problems, e.g., no variance in the outcome variable. However, when the analysis involves a categorical independent variable, and there is a small cell count in one of the categories, the occurrence of NA s might also be indicative of one of the categories not appearing in some of the bootstrap samples, leaving it out of the samples’ GLMs. The failed.bootstrap.samples element would then show the presence of NA s for that particular category.

To our knowledge, the possibility to easily investigate problematic bootstrap samples is not implemented in other software with bootstrapping procedures. This functionality makes the ClusterBootstrap package useful when applying the bootstrap to GLMs in general, even when there is no clustering in the data. For these applications, one could set clusterid to a unique identifier for each observation (i.e., each row in the data).

## Simulation study: comparison to mixed models

The guidelines for presenting the design of a simulation study as recommended by Skrondal (2000) is used to present the current Monte Carlo experiment.

### Statement of research problem

This experiment investigates the impact of omitting a random effect and adding a redundant random effect to LMM, and whether the use of GLMCB leads to more proper statistical inference. Usually, it is unknown to what extent the random effects structure has to be specified, and it is difficult to assess whether this is done properly. With GLMCB, there is no need for specification of random effects, making statistical inference with respect to the individual explanatory variables insusceptible to errors in this specification. The effects of sample size and ICC of the random slope will be part of the investigation. It will also be investigated whether there is a difference between balanced and unbalanced data at the level of the repeated measurements.

### Experimental plan and simulation

Data are simulated according to a LMM presented in Singer and Willett (2003, p. 184) that was fitted on the medication data described earlier. The model looks like

$$ Y_{ti} = \beta_{0} + \beta_{1}G_{i} + \beta_{2}T_{ti} + \beta_{3}G_{i}T_{ti} + U_{0i} + U_{1i}T_{ti} + \epsilon_{ti}, $$

with *Y*_*ti*_ being the outcome variable for person *i* at timepoint *t*, *G* being a group indicator (0 or 1), *T* being a time indicator, the random effects *U*_0*i*_ and *U*_1*i*_ being drawn from a multivariate normal distribution (specified below) and $\epsilon _{ti} \sim \mathcal {N}(0, 1229.93)$, as specified by Singer and Willett (2003). Values for *β*_1_ and *β*_2_ are constrained to zero, whereas *β*_0_ and *β*_3_ are set to the values 167.46 and 5.54, respectively. Between datasets, three factors were varied (details below):

1. Sample size: 16, 32, or 64 subjects;
2. ICC: .05, .30, or .50. The mixed model fitted on the original data in Singer and Willett (2003) reported an ICC of .05;
3. Balanced vs. unbalanced data regarding the number of measurement occasions.

To keep the correlation between the simulated random intercept and slope (*r* ≈−.33) intact, random effects are drawn from a multivariate normal distributions with mean vectors 0 and covariance matrices

$$ \begin{array}{@{}rcl@{}} \boldsymbol{\Sigma} &=& \left[\begin{array}{ll} 2111.33 & \\ -121.62 & 63.74 \end{array}\right], \left[\begin{array}{ll} 2111.33 & \\ -349.74 & 527.11 \end{array}\right] \mathrm{,} \\ &&\text{and} \left[\begin{array}{ll} 2111.33 & \\ -534.24 & 1229.93 \end{array}\right], \end{array} $$

for ICC = .05, .30, and .50, respectively. The distinction between balanced and unbalanced data is made as follows. For balanced data, each person is set to have four repeated measurements (*t* = {0,1,2,3}). In the unbalanced condition, the number of repeated measurements and the value of the time indicator at follow-up measurements are varied between subjects. Besides a measurement at timepoint *t* = 0, subjects are simulated to have one, two or three follow-up measurements, with integer values of *t* sampled from a uniform distribution in the range [1, 3]. In the following paragraphs, the distinction between balanced and unbalanced data will be referred to as the “balanced” condition.

### Estimation

For the LMMs, restricted maximum likelihood is used to obtain parameter estimates, using the BFGS algorithm within the nlme package (Pinheiro et al., 2014) in R (R Core Team, 2016). The fixed part of the fitted models all include the group and time variable, as well as their interaction. Within each dataset, the LMMs were operationalized in three forms, differing in the specification of the random effects:

1. The correctly specified LMM contains both the random intercept and random slope;
2. The underspecified LMM only contains the random intercept;
3. The overspecified LMM contains both simulated random effects, as well as an additional fixed and random effect for quadratic time.

The GLMCB models all contain the group and time variables, as well as their interaction. Each GLMCB is set to create 5000 balanced bootstrap samples, applying a 95% BCa confidence interval for the assessment of statistical significance as well as coverage of the simulated fixed effects.

### Replication

For each of the 18 *N* ×*ICC* ×balanced dataset configurations, the steps above are simulated 200 times. Within each of the simulations, GLMCB is performed, as well as the correctly specified, the underspecified and the overspecified LMM.

### Analysis of output

For all four models in every replication, the estimated regression coefficients (for GLMCB) or fixed effects (for LMM) $\hat {\beta }_{2}$ and $\hat {\beta }_{3}$ are saved, as well as their statistical significance. We chose for the focus on $\hat {\beta }_{2}$ and $\hat {\beta }_{3}$ because it provides insight in both type I error rates (for $\hat {\beta }_{2}$) and power (for $\hat {\beta }_{3}$). For GLMCB, it is assessed whether 0 falls within the 95%CI for each of the regression coefficients. For LMM, fixed effects are considered statistically significant when *p* < 0.05. Coverage of the true (i.e., simulated) coefficient in the confidence intervals is also assessed for these *β* s.

For *β*_2_ and *β*_3_, bias is calculated for each technique within each of the 200 simulations of each *N* ×ICC configuration. Type I error rate (*β*_2_ only), observed power (*β*_3_ only) and coverage rate (*β*_2_ and *β*_3_) are calculated within each technique as percentages of the 200 simulations of each of the configurations.

#### Bias

Within each *N* ×*ICC* ×balanced combination, bias values are calculated for each of the used techniques as

$$ \text{Bias} = \frac{1}{200} \sum\limits_{r=1}^{200}(\hat{\beta}_{r} - \beta). $$

#### Type I error rate

For every technique under investigation, the percentage of type I errors for *β*_2_ is calculated. For the GLMCB procedure, it is the percentage of the 200 simulations within which 0 falls outside the 95% CI. For LMM, the percentage of type I errors for *β*_2_ is defined as the percentage of 200 simulations in which *p* < 0.05.

#### Observed power

For GLMCB, the observed power of *β*_3_ is defined as the percentage of the simulations within which 0∉95*%* CI and the sign of the estimated effect is the same as the sign of the true effect (i.e., there is a statistically significant, positive estimated value). For LMM, it is the percentage of simulations in which *p* < 0.05, also with an equal sign of the estimated and the true effect.

#### Coverage rate

The coverage rate of GLMCB is the rate at which the true value *β* lies within the estimated 95*%* CI of $\hat {\beta }$. For LMM, 95*%* CIs are based upon the given *t* value with the appropriate degrees of freedom for each parameter, at permilles 25 and 975.

The four outcome measures are analyzed interpretatively, with the aid of graphs. To help interpretation, 95*%* CIs are calculated. For the quantitative bias statistics, nonparametric confidence intervals are constructed. For the remaining proportional outcomes, primarily, Agresti–Coull intervals are calculated (Agresti and Coull, 1998). However, especially in the overspecified LMMs, missing values might occur due to optimization problems. When, due to these missing values, the number of remaining indicators is 40 or less, Wilson intervals (Wilson, 1927) will be calculated, as recommended by Brown et al., (2001).

#### Results

**Fig. 1:**
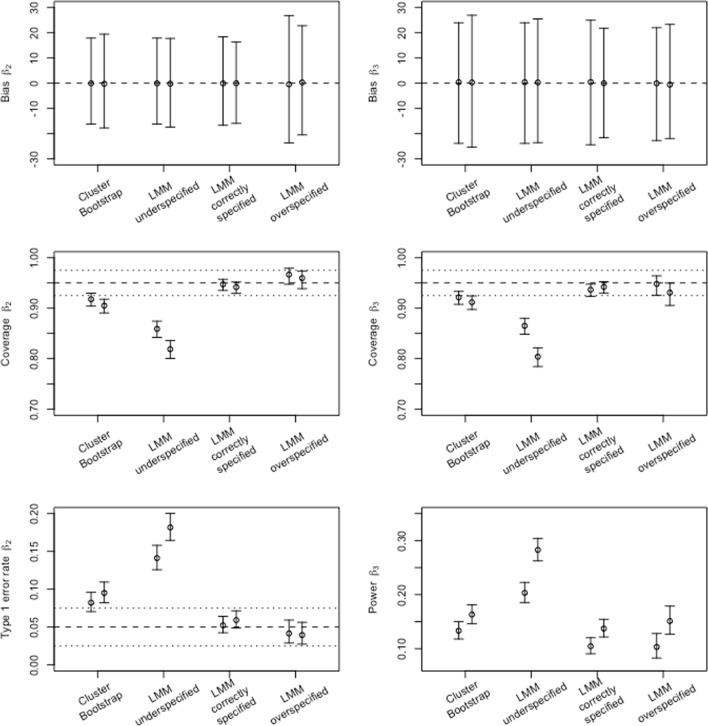
Summary of simulation results, aggregated over *N* and ICC conditions. The left-hand figures show the average bias and coverage values, as well as the type I error rate for *β*_2_. The right-hand figures show bias, coverage, and power averages for *β*_3_. For each of the four techniques within each subfigure, results are shown for the balanced (*left*) and the unbalanced (*right*) case. Confidence intervals (95%) are indicated with *error bars*. Conventional threshold values for bias (being 0), coverage (.95) and type I errors (.05) are indicated by *dashed horizontal lines*. *Dotted horizontal lines* depict .925 and .975 thresholds for coverage and .025 and .075 thresholds for type I error rate, as suggested by Bradley (1978)

The overall mean bias (averaged over all *N* ×ICC combinations) and CIs for GLMCB and the three LMMs are shown in Fig. 1, upper panel. It can be seen that there is no real difference in performance regarding bias, for both the balanced and the unbalanced case.

Figure 1 (middle panel) shows the coverage rates and corresponding CIs for both *β* s. As could be expected, the correctly specified LMM has .95 within its CI. It can also be seen that the cluster bootstrap performs only slightly below the 95% boundary. The overspecified LMM also performs well, and the underspecified LMM has much lower coverage. The underspecified LMM is inferior to the other techniques, and performs even worse with unbalanced data.

In the lower panel, Fig. 1 shows that underspecification of LMM leads to higher power, but also to higher type I error rates. Note that the higher power for the underspecified LMM does not necessarily bode well for underspecification of LMM. The higher type I error rates suggest that the baseline rejection rate of the null hypothesis is higher, which would lead to non-null effects to be detected more often by chance as well. The elevation of the type I error rate and power is stronger for the unbalanced case. Type I error rate for GLMCB is also slightly above the nominal level whereas the correctly specified and the overspecified LMM do well on both measures. Overall, in this simulation, power for *β*_3_ is low, presumably due to the sample sizes in our simulation being too small, given the effect size present in the data being simulated. Note that this is the case for the cluster bootstrap with GLM, as well as the correctly specified and overspecified LMMs.

More detailed graphs, for the 9 *N* × ICC combinations separately, can be found in Appendix A. In these graphs, it can be seen that regarding coverage and type I error rates, specifically CBGLM benefits slightly from larger samples. For *N* ≥ 32 the coverages and type I error rates are satisfactory for CBGLM. The benefit of larger samples for power is, expectedly, present for all techniques.

## Discussion

We introduced a new R package ClusterBootstrap for the analysis of the hierarchical data using GLMs using the cluster bootstrap. In contrast with the regular bootstrap, CBGLM resamples clusters of observations instead of single observations, for example all the repeated measurements within an individual. The package provides functionality for the main CBGLM analysis, incorporates different types of confidence intervals (parametric, percentile and BCa), has ample possibilities to explore the outcome, choose post hoc alternatives for parameters that were set in the initial analysis (level and type of confidence interval), and provides the user with methods of exploring bootstrap samples that had difficulties in fitting the proposed GLM. The current paper aims on the use of the ClusterBootstrap package for repeated measures, though it should be noted that the cluster bootstrap with GLM can be applied to other (cross-sectional) data as well, when there is a presence of clustering in the data (e.g., children within classes or patients within clinics). It should however be kept in mind that the resampling process should reflect the original sampling process. In our application for repeated measurements, subjects are gathered and each subject has a certain amount of repeated measurements. Analogous, the resampling procedure takes the complete set of repeated measurements of a specific subject into the bootstrap sample. If the original sampling process is different, this way of resampling may not be appropriate. For example, if one samples classes within schools, and subsequently samples some children (i.e., not all children) from each class, the bootstrap procedure should be adapted to not automatically include all gathered children within a class (i.e., observations within clusters). In this case, one could implement a two-step bootstrap, resampling children within resampled classes.

The main advantage of using CBGLM instead of other techniques that deal with hierarchical data, is the relatively low number of assumptions that have to be met for the outcome of the analysis to be valid. We compared CBGLM to three variations of LMM in a Monte Carlo experiment. In the first LMM variant, the random slope for the within-subject variable time was omitted, the second variant was correctly specified with a random intercept and the random slope, and the third variant had an extra fixed and random effect added for a quadratic time effect. It was shown that for coverage and type I error rate, the correctly specified LMM has a slight advantage over CBGLM, although for sample sizes of 32 or higher, the performance of CBGLM is satisfactory. The deteriorating effect of small samples on CBGLM’s performance is in line with earlier findings by Cameron et al., (2008) and McNeish (2017). The earlier finding of Dorman (2008) regarding the possible moderating effect of ICC strength on type I error rate with the omission of the regarding random effect, could not be replicated, and had no implications of the comparison of CBGLM to the three variations of LMM. Overall, the simulation study endorses the hypothesis that CBGLM outperforms underspecified LMMs.

There are two limitations to this study. First, in the Monte Carlo experiment, we used the specifications of a LMM to generate the data. This automatically makes the correctly specified variation of LMM superior to all other techniques applied. Though this can be seen as a form of self-handicapping in disadvantage of CBGLM, our aim was not to show that CBGLM could outperform LMM, but that knowing that the correct specification of LMM is problematic and that underspecification could very well invalidate the outcome of the analysis, CBGLM might be a relatively safe alternative. For larger sample sizes, the simulation study shows evidence for this. As an alternative to the correctly specified LMM being used for data generation, one could use additional variables in the generating process, which would not be included in the application of the techniques. This, however, would lead to the question how such a ”true” model could be formed. A second limitation is the application of the standard cluster bootstrap in the Monte Carlo experiment, although there are suggestions in the literature that for smaller samples, the so-called wild cluster bootstrap-*t* performs better (Cameron et al., 2008; McNeish, 2017). The wild cluster bootstrap-*t* is, however, not yet available in the ClusterBootstrap package. As the development of this package is an ongoing process, the addition of this option is planned for a future release. Other plans for future releases of the package are the implementation of the predict() command to support model predictions and an expansion to the penalized-likelihood framework. Implementing penalization in the cluster bootstrap would be particularly interesting, as it may offer a convenient means of dealing with separation in classification models for which the ClusterBootstrap package already offers investigation opportunities. To which extent the cluster bootstrap performs well when bias is introduced to the parameter estimates (i.e., bias towards zero) is an opportunity for further research. Our simulation ________ study suggests that the statistical power of CBGLM is comparable to the correctly specified LMM, which could mean that sample size calculations for LMM are appropriate for CBGLM as well. Further research is needed to investigate the required sample sizes under different circumstances (e.g., different effect sizes, power levels, numbers of repeated measurements confidence interval widths).

## Figures and Tables

**Fig. 2:**
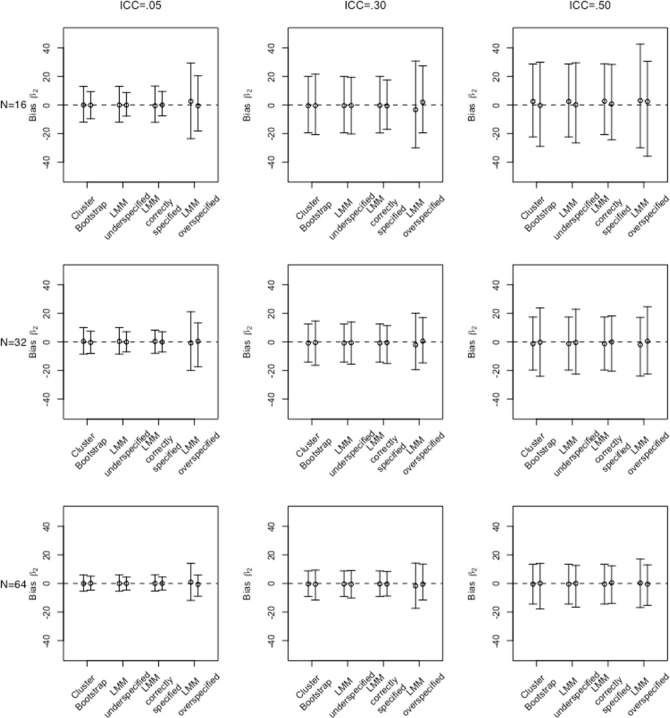
Bias for *β*_2_, for all *N* ×ICC combinations. For each of the four techniques within each subfigure, results are shown for the balanced (*left*) and the unbalanced (*right*) case. Confidence intervals (95%) are indicated with *error bars*

**Fig. 3:**
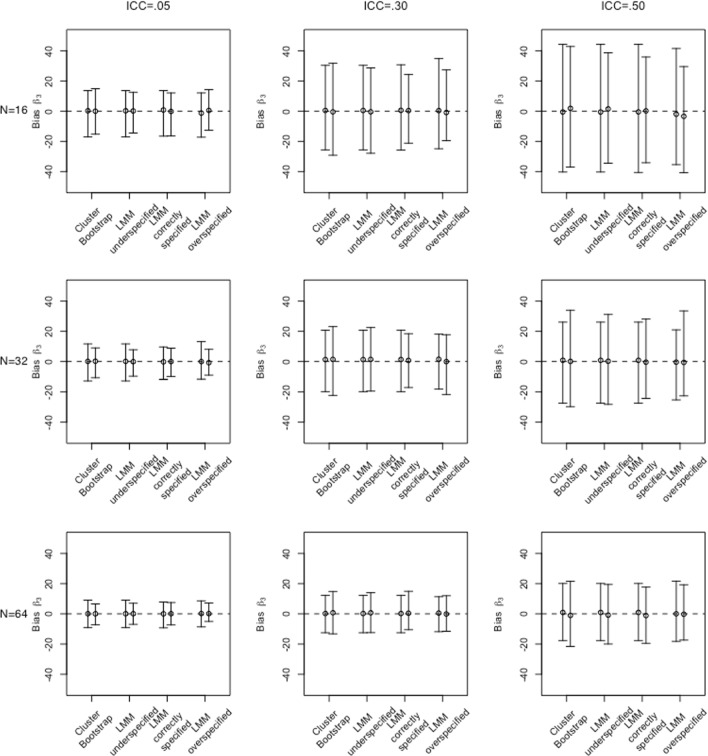
Bias for *β*_3_, for all *N* ×xICC combinations. For each of the four techniques within each subfigure, results are shown for the balanced (*left*) and the unbalanced (*right*) case. Confidence intervals (95%) are indicated with *error bars*

**Fig. 4:**
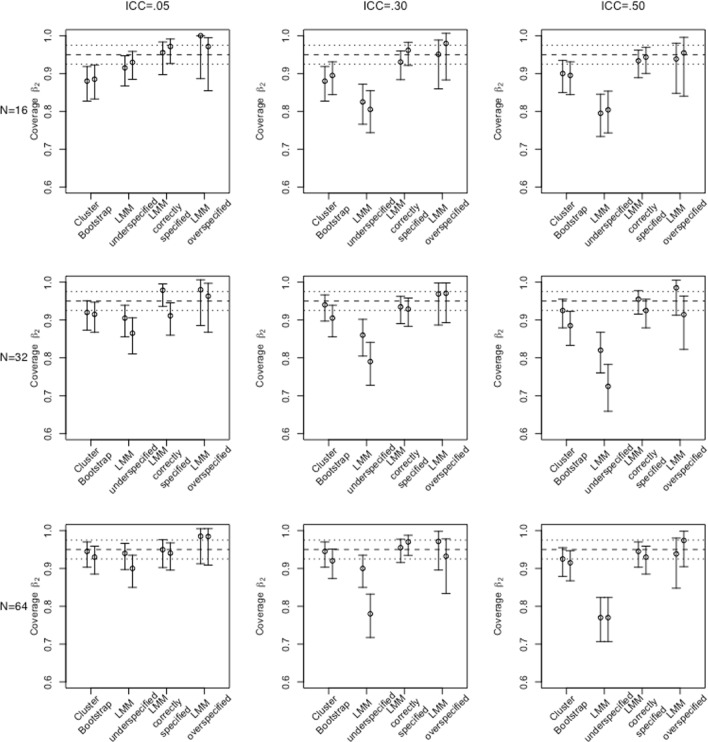
Coverage for *β*_2_, for all *N* ×ICC combinations. For each of the four techniques within each subfigure, results are shown for the balanced (*left*) and the unbalanced (*right*) case. Confidence intervals (95%) are indicated with *error bars*. The conventional threshold of 95% is indicated by *dashed horizontal lines* and the 92.5% and 97.5% levels are depicted by *horizontal dotted lines*

**Fig. 5:**
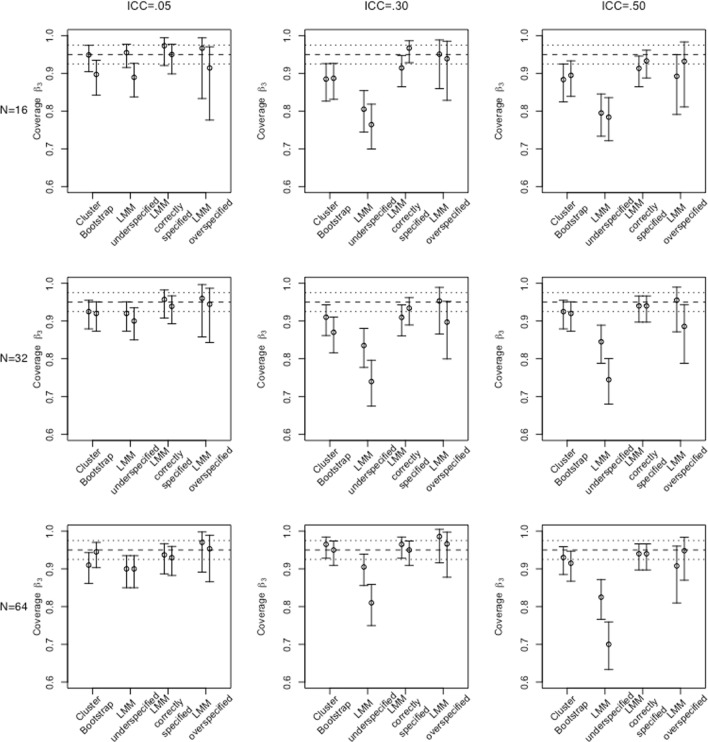
Coverage for *β*_3_, for all *N* ×ICC combinations. For each of the four techniques within each subfigure, results are shown for the balanced (*left*) and the unbalanced (*right*) case. Confidence intervals (95%) are indicated with *error bars*. The conventional threshold of 95% is indicated by *dashed horizontal lines* and the 92.5% and 97.5% levels are depicted by *horizontal dotted lines*

**Fig. 6:**
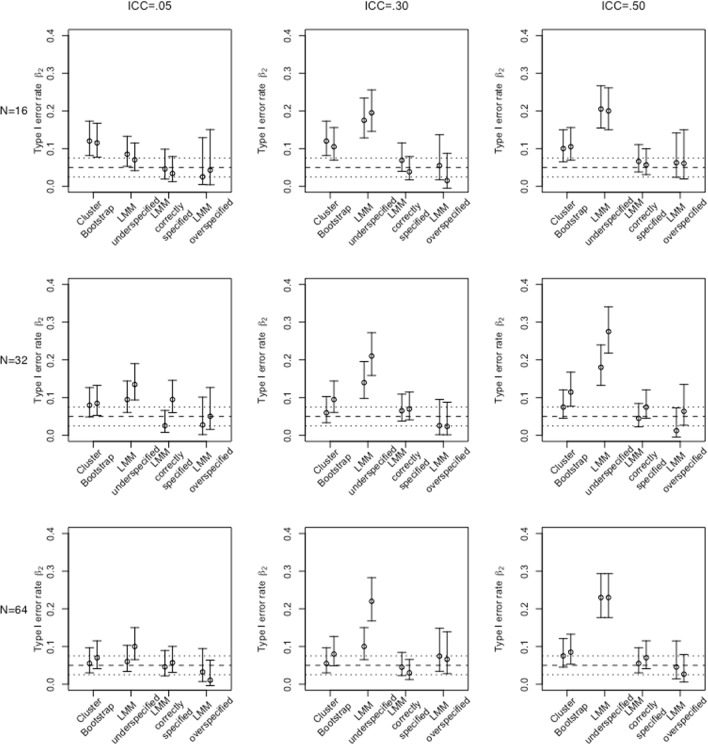
Type 1 error rate for *β*_2_, or all *N* ×ICC combinations. For each of the four techniques within each subfigure, results are shown for the balanced (*left*) and the unbalanced (*right*) case. Confidence intervals (95%) are indicated with *error bars*. The conventional threshold of 5% is indicated by *dashed horizontal lines* and the 2.5% and 7.5% levels are depicted by *horizontal dotted lines*

**Fig. 7:**
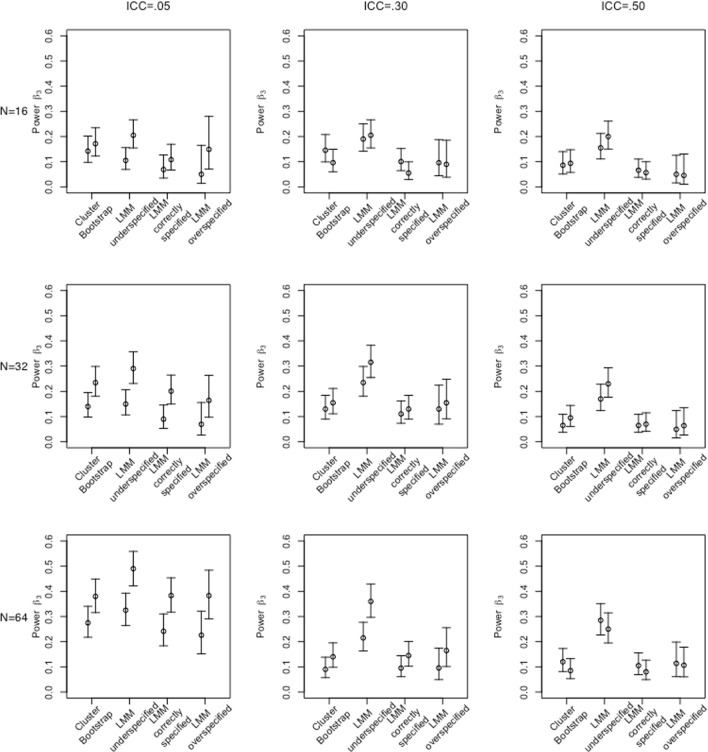
Power for *β*_3_, or all *N* ×ICC combinations. For each of the four techniques within each subfigure, results are shown for the balanced (*left*) and the unbalanced (*right*) case. Confidence intervals (95%) are indicated with *error bars*. The conventional threshold of 80% is not depicted
